# Institutional Notes and News

**Published:** 1911-07-15

**Authors:** 


### Institutional Notes and News.

**The Salford Hospital Extensions Opened.**

On Thursday last week the formal opening of the new extensions to the Salford Royal Hospital, by the Right Hon. Lord Shuttleworth, Lord-Lieutenant of Lancashire, took place. The new extensions comprise the borough's memorial to the late King Edward, in the shape of a new wing to the hospital, called the "King Edward VII. Memorial Wing," on the east side of the hospital, and which contains forty-five beds. There are also new ward wings, an additional storey over the administrative buildings to provide sleeping accommodation for the domestic servants, nurses' home, and two operating theatres above the central block of the new ward building. The new wards consist of an east and north wing, in each of which are three wards, 56 ft. by 26 ft., of fourteen beds. This provides ninety beds in the extension, including the private wards. In the basement of this block is a clinical laboratory. The new wards communicate with the old by cross-ventilated corridors across the central yard. The out-patients' hall is 48 ft. by 36 ft. On the north side is a separate egress; on the east is the dispensary, with a pharmaceutical laboratory and store rooms. The south side has seven consulting and examination rooms, and on the west are the accident room, the small operating theatre, and anæsthetising room. The hall has accommodation for two hundred. The original hospital building is now the new administrative block with accommodation for the resident medical and surgical staff on the first floor, and the matron's quarters are in direct communication with the nurses' home and servants' quarters. A new entrance hall has been built between the nurses' home and this block. The hospital, as completed, contains 209 beds, but for the present it has been decided to open 174 beds only. This will leave two of the smaller wards in the old buildings vacant till further financial support is forthcoming. The extension fund, including the King Edward Memorial Fund, now amounts to £58,083, and the Board appeals for the remaining sum of £11,917, so that there may be no need to draw on the invested capital.

At the ceremony of opening Sir William Mather, president of the institution, was in the chair. There were also present the Mayor of Salford (Mr. Alderman Phillips), Mr. Frederick Burton (treasurer), Mr. Lawrence Pilkington (chairman of the Board of Management), Mr. Edward G. Wood (chairman of Extension Committee), Mr. Alderman Snape (ex-Mayor), Mr. Edward Hopkinson (chairman of Building Committee), Sir Edward Donner, Sir George W. Agnew, Mr. Alderman Frankenberg, Dr. A. M. Edge, Mr. Alderman Linsley, and Mr. L. C. Evans (Town Clerk).

Sir William Mather reminded his hearers of the growth of the hospital from its small beginnings in 1830, and complimented the men who have been most prominent in raising the needful funds for the extensions and in bringing the extension scheme to a successful end. Many Salford people besides supporting contributed to the help of Manchester institutions to a much greater extent, he said, than Manchester people support Salford schemes.

Lord Shuttleworth said that one of his greatest privileges was to take a humble part in the opening of new buildings connected with one or another of the great hospitals in this county and, as Sir William Mather had said, help to give a good send-off to the new enterprise. He had been very much struck during the last three years, with the remarkable number of efforts for the extension or improvement of hospitals, and he desired to congratulate the Salford people on the accomplishment of this noble work. Lord Shuttleworth recalled his father's association with Manchester and Salford and the part his father, a physician, was called upon to play in dealing with the cholera scourge which swept over Manchester in 1831. He also spoke of the extreme desirability of municipal corporations thoroughly clearing away those places to which the horrible word "slum" was attached.

Mr. Lawrence Pilkington, after pointing out that it is much easier to build a hospital than to maintain it, asked for an increased number of subscribers. When the visitors went round and through the extensions he was confident they would admit that their money had been wisely spent.

Mr. Edward G. Wood, who as chairman of the Extension Committee was able to collect £50,000 in four years, offered the Committee's warmest and heartiest thanks to all the contributors to the extension fund. Mr. Wood said he believed that the union of Manchester and Salford would come about in the near future, when one of the best assets will be the Salford Royal Hospital.

**Patients' Day at the Royal Free Hospital.**

The new shelter and other improvements for the patients at the Royal Free Hospital, Gray's Inn Road, have only awaited the conclusion of the Coronation festivities for their inauguration. Princess Christian, the hospital president, arranged to inaugurate them last week, for which ceremony the quadrangle of the hospital forms a most charming background. The occasion, however, was intended to be a patients' occasion in particular, the hospital board having determined to commemorate the Coronation by giving a tea to the patients in the wards. The patients had only to await the conclusion of the ceremony in the quadrangle for the Princess to be among them, and her Royal Highness was probably aware that her visit was less a public than a patients' function, which was calculated to bring them into closer touch with the president of their institution than is possible at such merely formal occasions as the unveiling of a tablet or the opening of a ward. Coronation mugs were given to the patients. The shelter was paid for out of the Louisa Lady Goldsmid grant.

**New Site of the Central Ophthalmic Opened.**

A distinguished company assembled on Wednesday last week to take part in the ceremony of laying the foundation-stone of the new building of the Central London Ophthalmic Hospital. Removal has been rendered necessary, as The Hospital pointed out on July 1 (page 341), by the expiration of the lease of the existing premises, which, with extensions from time to time, have done duty for seventy years past, and a new site has been found in Judd Street, St. Pancras. H.R.H. the Duchess of Albany was received by the President of the hospital, the Right Hon. the Earl of Stradbroke, the chairman and members of the committee, and senior members of the surgical and medical staff. The Earl of Stradbroke related how the demands upon the hospital had grown, and that in this new building they would be able more efficiently to cope with the still increasing number of patients. No fewer than 453,484 separate patients had attended for treatment, whilst during last year there were 12,587 out-patients and 417 in-patients. The scheme had been approved by King Edward's Hospital Fund, from which £1,000 had been granted towards the cost of the new hospital. The total cost involved was

£20,000, of which £12,000 had so far been subscribed. His Lordship concluded : " The value of the objects of the charity is beyond all comparison to the smallness of the amount which is needed to instal it in its new home." Mr. John Ladds, the architect, then handed an inscribed silver trowel to her Royal Highness, who proceeded to lay the foundation-stone and received purses, the Secretary, Mr. H. R. S. Druce, announcing that the amount so subscribed came to £375. Captain Jessel, M.P., proposed a vote of thanks to the Duchess of Albany, and congratulated the committee on the excellent site it had acquired, and predicted that the hospital would now enter upon a new era of greater service to the necessitous poor. Mr. George Miles, deputy-chairman of the committee, seconded the vote of thanks, to which her Royal Highness replied and made a critical inspection of the plans.

